# Changes in prescription patterns and drug costs following the launch of generic pemetrexed products: a retrospective study in Japan

**DOI:** 10.1186/s40780-026-00570-6

**Published:** 2026-04-02

**Authors:** Toshikazu Ichie, Daisuke Suzuki, Shuji Yamashita, Kunio Naruse, Hideki Hayashi

**Affiliations:** 1 Department of Pharmacy, Kainan Hospital, 396 Minami-Honden, Maegasu-cho, Yatomi, Aichi 498-8502 Japan; 2 Laboratory of Community Pharmaceutical Practice and Science, Gifu Pharmaceutical University, 1- 25-4 Daigakunishi, Gifu, 501-1196 Japan

**Keywords:** Pemetrexed, Generic drug, Drug costs, Medical economics, Administrative claims

## Abstract

**Background:**

Pemetrexed (PEM) is an antimetabolite antineoplastic agent that has been available in generic form in Japan since June 2021. As a hazardous drug (HD), the use of a closed-system transfer device (CSTD) is recommended. Some generics, including 800-mg vials, are not available for the original product; however, changes in prescribing patterns following the introduction of generics remain unclear. This study examined the prescription volume and drug costs of PEM before and after the launch of generics.

**Methods:**

Clinical data were analyzed using the MDV analyzer^®^ (Medical Data Vision Co., Ltd.). Patients who received PEM between June 2020 and May 2023 were enrolled. Study outcomes included the number of prescriptions, dose per prescription, drug costs (yen), and vials used. Data were categorized into original PEM, generics without 800-mg vials, and generics with 800-mg vials.

**Results:**

A total of 89,078 prescriptions were analyzed. In the last 3 months of the study, prescription counts (proportions) were 966 (14.8%) for original PEM, 404 (6.2%) for generics without 800-mg vials, and 5,155 (79.0%) for generics with 800-mg vials. The mean prescribed doses were 773.2 mg, 789.6 mg, and 777.8 mg, respectively. The mean drug costs were 193,683 yen, 84,583 yen, and 78,770 yen, respectively. The average numbers of vials per prescription, calculated from the total number of vials and prescriptions, were 3.4, 3.5, and 2.3, respectively, indicating that generics with 800-mg vials were associated with approximately one fewer vial per prescription compared to other groups.

**Conclusions:**

After the introduction of generics, a shift in prescription patterns was observed, with an increasing proportion of products with 800-mg vials. Although mean prescribed doses were similar across groups, generics with 800-mg vials appeared to be more cost-effective and utilized fewer vials per prescription within this study population. The reduction in the number of vials suggests a potential decrease in CSTD use, which could contribute to lowering material costs. These results provide important insight for future drug development and for strategies aimed at reducing healthcare expenditures, including material costs.

## Background

Pemetrexed (PEM) is classified as an antimetabolite and antineoplastic drug. In Japan, it has been approved for the treatment of malignant pleural mesothelioma as well as unresectable, advanced, or recurrent non–small cell lung cancer (NSCLC). PEM is recommended as an option for first-line therapy, maintenance therapy, and second-line therapy in non-squamous NSCLC. For advanced lung cancer without driver gene alterations—such as epidermal growth factor receptor mutations, anaplastic lymphoma kinase rearrangements, or ROS proto-oncogene 1, receptor tyrosine kinase rearrangements—combination therapy with platinum-based agents is considered the standard treatment [1]. According to the package insert, the recommended dose and administration consist of 500 mg/m^2^ of body surface area administered once every cycle, with a minimum of 20 days between doses, and dose reduction as necessary based on the patient’s condition. With respect to the history of PEM in Japan, the original formulation was launched in 2007; however, only 500-mg lyophilized formulations were available. A 100-mg formulation was later introduced in September 2009 [2]. In Japan, PEM is considered one of the key drugs for NSCLC treatment. Given this dosage regimen, it is often necessary to combine multiple strengths and vials for administration.

Since June 2021, generics of PEM have been available in Japan. In addition to being less expensive than the original product, the introduction of generic PEM has a significant economic impact on reducing healthcare costs, given that PEM is a high-priced medication. Furthermore, while most generics are typically available only in the same formulations and strengths as the original product, some generic PEM products offer formulations that are not available for the original product, such as liquid formulations and 800-mg vials. The use of liquid formulations eliminates the need for reconstitution, whereas the availability of 800-mg vials reduces the number of vials required per patient. Consequently, these products are expected to be associated with shorter preparation times. Furthermore, because PEM is classified as a hazardous drug (HD) [3] and the use of CSTDs is recommended in the Japanese guidelines [4], prescriptions using 800-mg vials may also lead to a reduction in the amount of preparation equipment required during compounding.

Reports comparing original and generic PEM formulations in Japan have shown the use of liquid formulations, including 800-mg vials, results in no difference in safety, while reducing preparation time and drug costs; however, these findings were based on a single-center study and examined only one formulation [5]. In addition, regarding the introduction of new vial sizes, revisions to available vial sizes for various drugs, including PEM, can alter drug costs; however, previous studies were based on cost estimates derived from administered doses in clinical practice [6, 7]. Previously, we reported that following the introduction of a lower-dose vial size for ipilimumab, changes in prescribed volumes and drug costs were observed in real-world practice [8]. Nevertheless, the impact of the launch of generic PEM formulations on clinical practice has not yet been evaluated. Since PEM is widely administered to many patients, it may be important for medical institutions to consider not only drug costs but also value-added features, such as shorter preparation times and potential changes in CSTD utilization efficiency. These characteristics are considered distinct from those associated with the introduction of conventional generics. Furthermore, while single-center reports exist, there is limited research comprehensively investigating the impact of diverse formulation strengths on real-world prescription patterns and economic outcomes using a nationwide clinical database.

In this study, medical records from the MDV Analyzer^®^ (Medical Data Vision Co., Ltd.) were analyzed to examine the changes in prescribed volumes and drug costs of PEM formulations following the introduction of generics in Japan across multiple medical institutions and formulations. The primary objective of this study was to clarify the changes in prescription patterns and drug costs before and after the launch of generic PEM in Japan. As a secondary objective, we aimed to explore the association between the introduction of 800-mg vials and liquid formulations and healthcare expenditures, as well as discuss their potential implications for CSTD utilization based on the number of vials used.

## Methods

### Study design

This retrospective analysis was conducted using medical records from the MDV Analyzer^®^ (Medical Data Vision Co., Ltd.). MDV Analyzer^®^ is a web-based service for analyzing medical databases in Japan. The database includes inpatient and outpatient claims data, as well as diagnosis procedure combination (DPC) data collected from domestic medical institutions that consented to secondary use of their data. Anonymization was conducted at each institution using dedicated tools before data submission. The database enables the analysis of clinical data from April 2010 onward. As of November 2024, it contained clinical data from 52.93 million patients (including deceased individuals) accumulated from 572 medical institutions nationwide [9].

### Study period

The study period spanned 36 months, from June 2020 to May 2023, and encompassed the 12 months before and the 24 months after the launch of the generic PEM.

### Study population

Eligible medical institutions were limited to those that had continuously provided clinical data to Medical Data Vision Co., Ltd. throughout the study period (June 2020 to May 2023), as of July 2024. This inclusion criterion was applied to ensure a stable institutional cohort and to eliminate temporal fluctuations caused by the expansion of the database itself. The study population included all patients who were prescribed PEM formulations at these institutions. Patients who were prescribed multiple products of PEM formulations on the same billing date were excluded. For individual patients, duplicate counting did not occur if they had multiple visits or attended multiple departments within the same institution that provided data to Medical Data Vision Co., Ltd.; however, if the same patient visited different institutions that contributed data to Medical Data Vision Co., Ltd., they were considered separate patients.

### Study variables

The study variables included the number of institutions, number of patients, number of prescriptions, vial strength, prescribed dose (potency), drug cost, and number of vials prescribed. These variables were analyzed as absolute counts to describe the actual volume of generic substitution and the associated changes in drug expenditure within the target population. Temporal trends were evaluated at 3-month intervals (quarterly). The drug prices during each period are listed in Table 1. Due to the structural constraints of the MDV analyzer^®^, because all of the results were based on actual claims data, drug costs were calculated based on the drug prices at the time of billing. For cases in which residual amounts were also billed, both the prescribed dose and drug costs included the residual amounts. Detailed clinical parameters not captured in the claims data, such as patient weight, body surface area, and underlying diseases, were not included. Additionally, specific institutional billing practices—such as whether discarded residues were billed—and the timing of specific vial strength adoption by each institution could not be verified.

**Table 1:** Target drugs and their prices (Yen)

Number	Classification	Dosage form	Standard	Drug prices (Yen)			
				period			
				2020/06–2021/03	2021/04–2022/03	2022/04–2023/03	2023/04–2023/05
Original product	Original product	lyophilized	100 mg	45,067	45,048	31,134	28,671
			500 mg	188,457	188,457	123,462	112,999
Generic drug 1	Generics	lyophilized	100 mg	-	16,747	13,656	11,346
			500 mg	-	70,061	56,970	47,552
Generic drug 2	Generics	lyophilized	100 mg	-	16,747	13,656	11,346
			500 mg	-	70,061	63,053	47,552
Generic drug 3	Generics	lyophilized	100 mg	-	16,747	13,656	11,346
			500 mg	-	70,061	56,970	47,552
Generic drug 4	Generics	liquid	100 mg	-	16,747	13,656	11,346
			500 mg	-	70,061	56,970	47,552
Generic drug 5	Generics	liquid	100 mg	-	16,747	13,656	11,346
			500 mg	-	70,061	56,970	47,552
Generic drug 6	Generics	lyophilized	100 mg	-	16,747	13,656	11,346
			500 mg	-	70,061	56,970	47,552
			800 mg	-	106,410	85,510	70,743
Generic drug 7	Generics	lyophilized	100 mg	-	16,747	13,656	11,346
			500 mg	-	70,061	56,970	47,552
			800 mg	-	106,410	85,510	70,743
Generic drug 8	Generics	lyophilized	100 mg	-	16,747	13,656	11,346
			500 mg	-	70,061	56,970	47,552
			800 mg	-	106,410	85,510	70,743
Generic drug 9	Generics	liquid	100 mg	-	16,747	13,656	11,346
			500 mg	-	70,061	56,970	47,552
			800 mg	-	106,410	85,510	70,743
Generic drug 10	Generics	liquid	100 mg	-	16,747	13,656	11,346
			500 mg	-	70,061	56,970	47,552
			800 mg	-	106,410	85,510	70,743

Dates are presented in the YYYY/MM (Year/Month) format

### Cost-saving simulation

To estimate the potential economic impact of introducing generic PEM formulations, a cost-saving simulation was performed under a 100% substitution scenario within the study population. This model assumed that all prescriptions for PEM formulations during the target period (June 2022 to May 2023) were completely replaced by either generics without 800-mg vials or generics with 800-mg vials.

The estimation process was as follows: First, the total drug expenditure and the total number of prescriptions for each group were obtained from the MDV analyzer^®^, and the mean drug cost per prescription was calculated by dividing the total expenditure by the number of prescriptions. Next, the difference in the mean drug cost between the original product and each generic group was determined. The estimated annual reduction in drug expenditure was then derived by multiplying this difference by the total cumulative number of PEM prescriptions identified during the same period. Regarding the drug costs obtained from the MDV analyzer^®^, the analysis was conducted using data based on the official drug prices at the time of billing, in accordance with the system’s specifications.

### Statistical analysis

All study variables were evaluated using descriptive statistical methods. Due to the system specifications of the MDV analyzer^®^, when extracting data limited to medical institutions that provided continuous claims data throughout the study period, the total prescribed dose (potency) per drug could be calculated for each period (at 3-month intervals [quarterly]), but the distribution of doses per individual prescription could not be determined. Consequently, the prescribed dose was evaluated only as a mean value calculated from the total prescribed volume and the number of prescriptions. Similarly, since the distribution of the number of vials per prescription for each period could not be verified, the average number of vials per prescription was presented only as a mean value derived from the total number of vials and the total number of prescriptions across the entire study period. In addition, detailed patient-level parameters such as height, weight, and administration intervals were not available in the claims data and therefore could not be adjusted for in the analysis. As a result, the findings should be interpreted as descriptive summaries of prescribing patterns observed in routine clinical practice under real-world conditions. By restricting the analysis specifically to the population of PEM users rather than calculating utilization rates relative to the entire cancer population, this study focuses on describing product substitution dynamics and associated cost differences following generic entry.

## Results

**Fig. 1:**
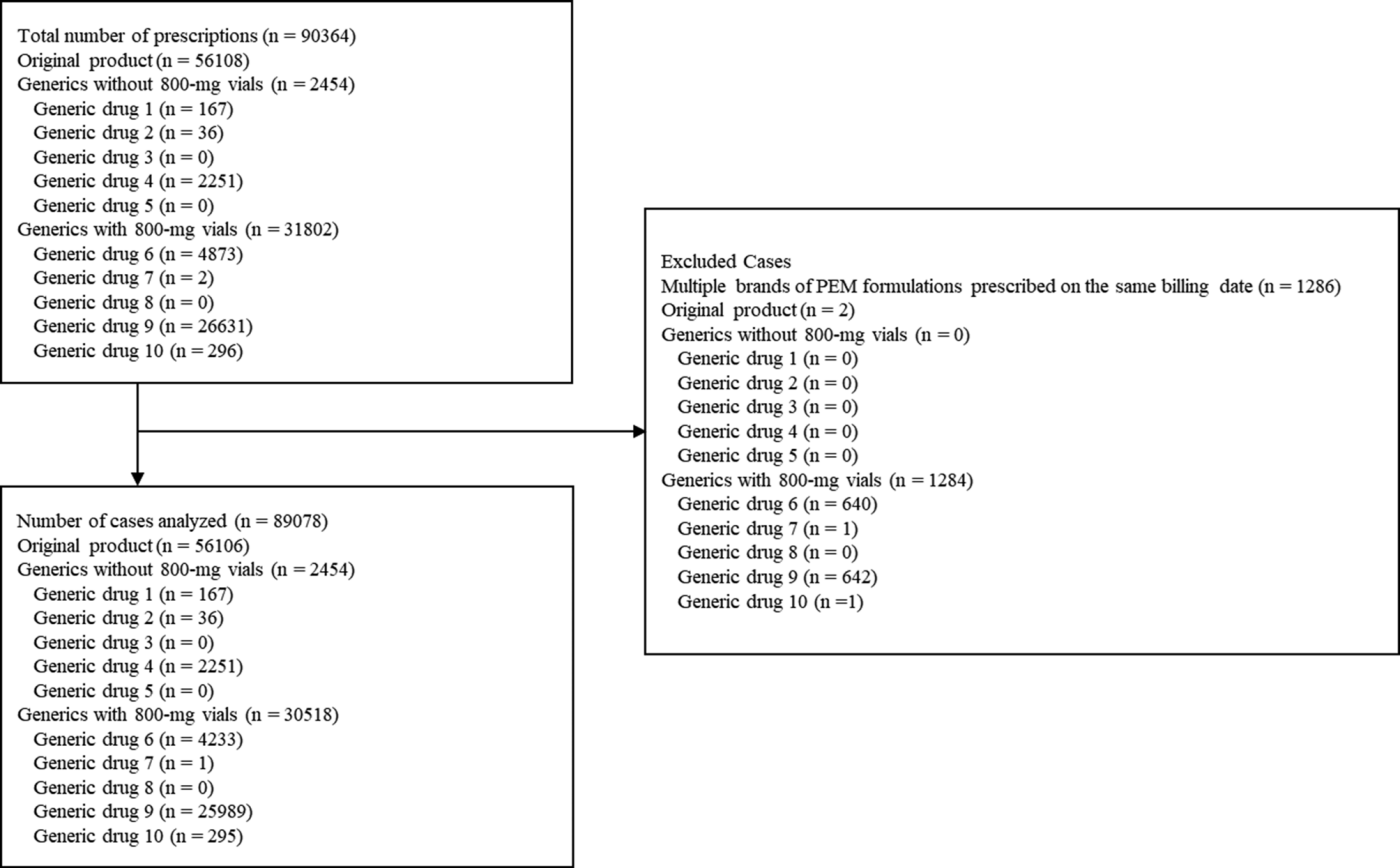
Patient selection flowchart

During the study period, a total of 420 institutions provided clinical data to Medical Data Vision Co., Ltd., covering 19,819,256 patients. The study population is illustrated in Fig. 1. Of the 90,364 total PEM prescriptions, 1,286 cases in which multiple products were prescribed on the same day, were excluded, thus resulting in 89,078 prescriptions included in the analysis. No prescriptions for “Generic drug 3,” “Generic drug 5,” or “Generic drug 8” were identified at any institutions during the study period. Patient backgrounds by formulation are listed in Table 2. In a comparison of generics, those with 800-mg vials were prescribed at more institutions and to more patients, and accounted for more prescriptions compared with those without 800-mg vials. Similarly, liquid generics were prescribed at more institutions, for more patients, and in greater numbers compared with lyophilized generics. Regarding patient characteristics across all formulations, the majority of prescriptions were for male patients, and approximately 70–80% of the patients were in their 60s and 70s.

**Table 2:** Patient characteristics by target drug

	Original product		Generics without 800-mg vials						Generics with 800-mg vials							
			Generic drug 1		Generic drug 2		Generic drug 4		Generic drug 6		Generic drug 7		Generic drug 9		Generic drug 10	
Number of prescriptions, n	56,106		167		36		2251		4233		1		25,989		295	
Number of institutions, n	263		2		3		18		34		1		142		6	
Number of patients, n	8531		32		11		368		733		1		4256		44	
Sex																
male, n (%)	5570	(65.3%)	18	(56.3%)	7	(63.6%)	247	(67.1%)	456	(62.2%)	0	(0%)	2691	(63.2%)	24	(54.5%)
female, n (%)	2961	(34.7%)	14	(43.8%)	4	(36.4%)	121	(32.9%)	277	(37.8%)	1	(100%)	1565	(36.8%)	20	(45.5%)
by age																
～49, n (%)	395	(4.6%)	1	(3.1%)	0	(0%)	12	(3.3%)	29	(4.0%)	0	(0%)	192	(4.5%)	1	(2.3%)
50～59, n (%)	1016	(11.9%)	6	(18.8%)	2	(18.2%)	46	(12.5%)	79	(10.8%)	0	(0%)	492	(11.6%)	4	(9.1%)
60～69, n (%)	2411	(28.3%)	8	(25.0%)	5	(45.5%)	110	(29.9%)	200	(27.3%)	0	(0%)	1165	(27.4%)	13	(29.5%)
70～79, n (%)	3919	(45.9%)	15	(46.9%)	4	(36.4%)	166	(45.1%)	343	(46.8%)	1	(100%)	1974	(46.4%)	20	(45.5%)
80～89, n (%)	786	(9.2%)	2	(6.3%)	0	(0%)	34	(9.2%)	81	(11.1%)	0	(0%)	426	(10.0%)	6	(13.6%)
90～, n (%)	4	(0%)	0	(0%)	0	(0%)	0	(0%)	1	(0.1%)	0	(0%)	7	(0.2%)	0	(0%)

**Fig. 2:**
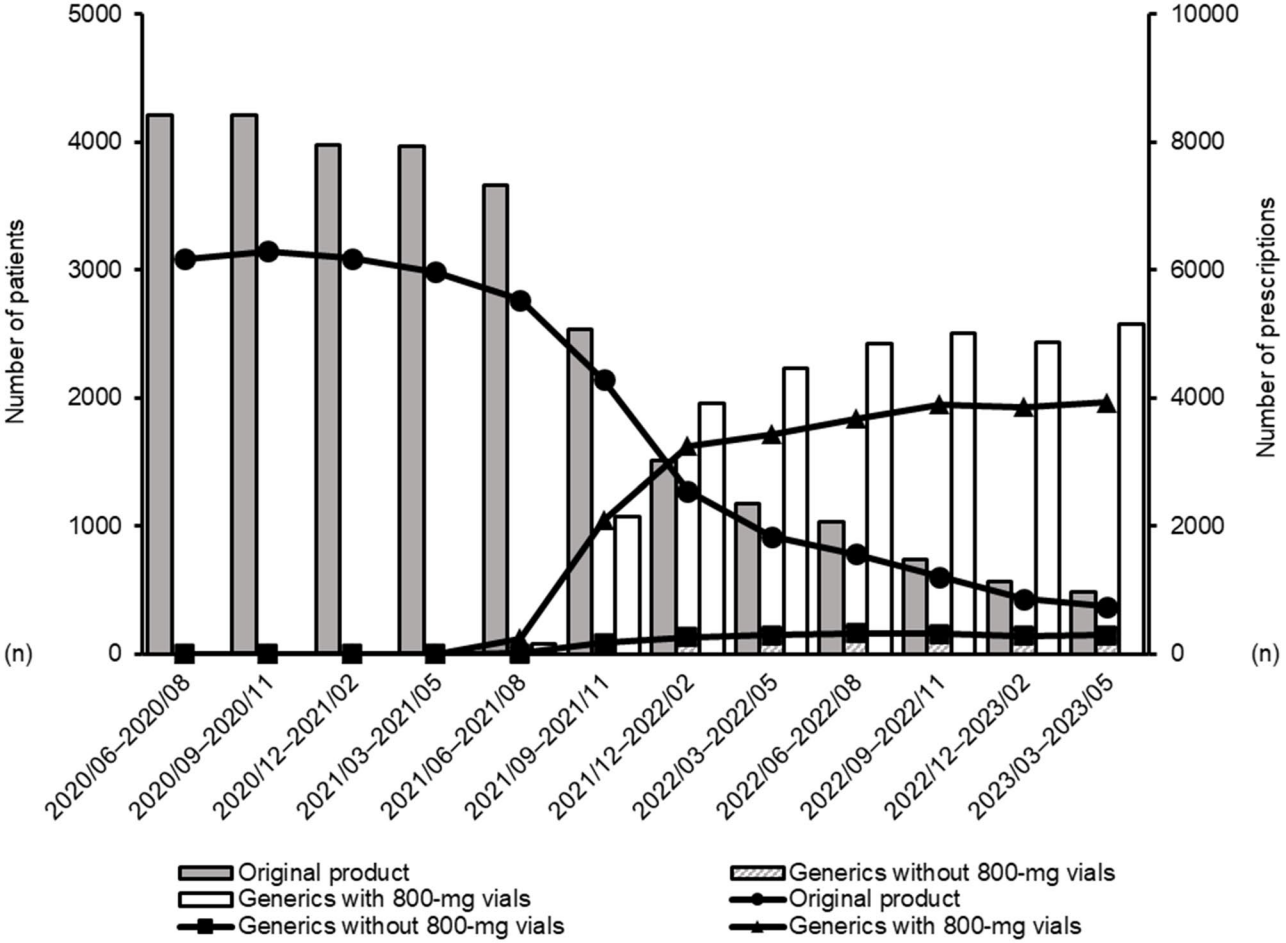
Temporal trends in the number of patients and prescriptions. Each data point represents an aggregate of a 3-month period (quarterly analysis). Dates on the horizontal axis are presented in the YYYY/MM (Year/Month) format

**Fig. 3:**
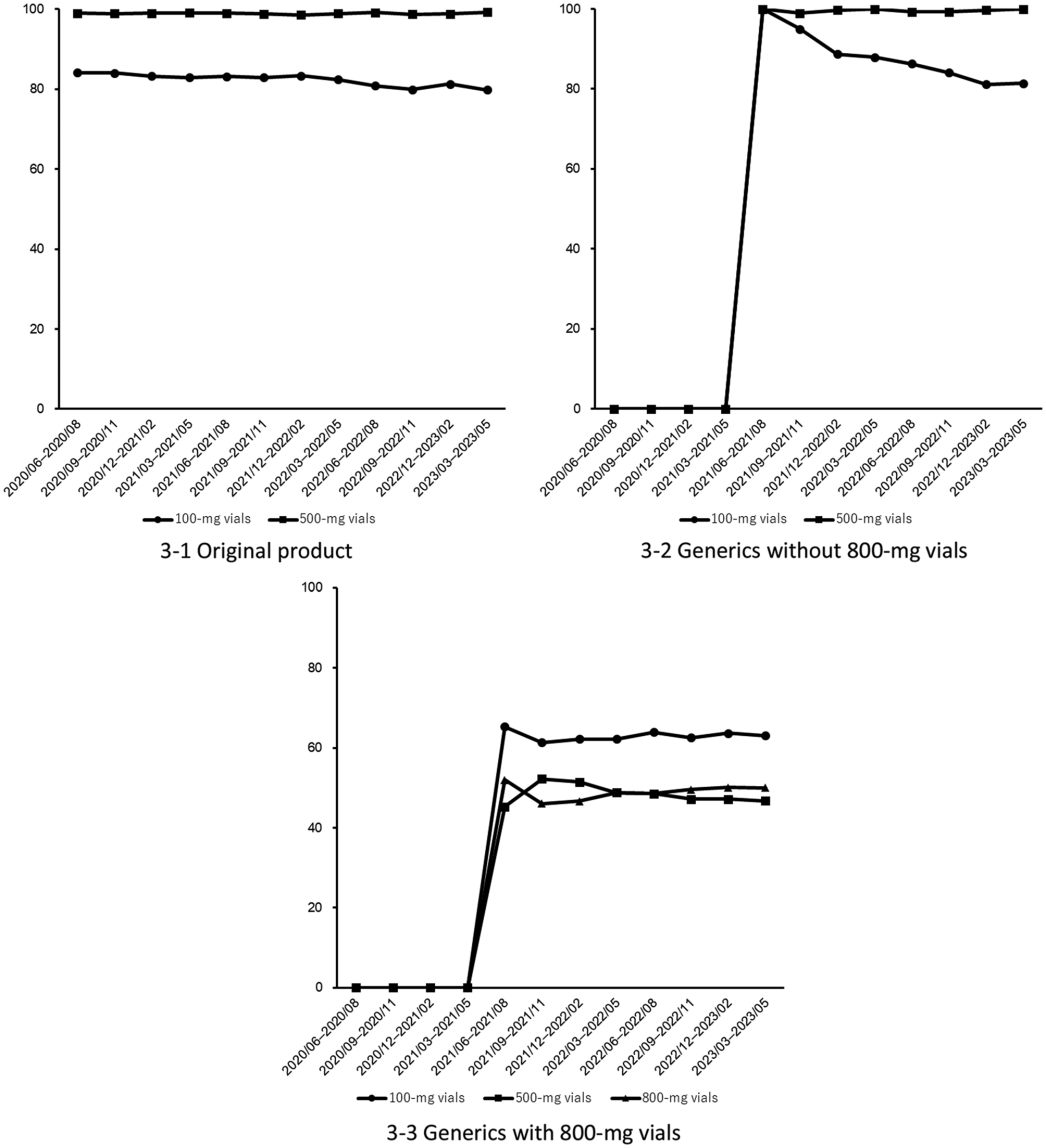
Temporal trends in the prescription proportions by dosage strength. 3 − 1 Original product: Each data point represents an aggregate of a 3-month period (quarterly analysis). Dates on the horizontal axis are presented in the YYYY/MM (Year/Month) format. 3-2 Generics without 800-mg vials: Each data point represents an aggregate of a 3-month period (quarterly analysis). Dates on the horizontal axis are presented in the YYYY/MM (Year/Month) format. 3-3 Generics with 800-mg vials: Each data point represents an aggregate of a 3-month period (quarterly analysis). Dates on the horizontal axis are presented in the YYYY/MM (Year/Month) format

When the target drugs were classified into three groups, including the original product, generics without 800-mg vials, and generics with 800-mg vials, the temporal trends in the number of patients and prescriptions every three months are shown in Fig. 2. After generics were introduced, the number of patients and prescriptions increased over time, with generics containing 800-mg vials representing the largest fraction at the end of the study period. The proportions of prescriptions by vial strength within each group are shown in Fig. 3. For the original product and generics without 800-mg vials, the proportion of 100-mg vials remained in the range of 70%–80%, and that of 500-mg vials within 90%–100% toward the end of the study period. In contrast, for generics with 800-mg vials, the proportion of 100-mg vials was approximately 60%, and that of 500-mg vials was 40%–50%. The proportion of 800-mg vials plateaued at approximately 40%–50% toward the end of the study period.

**Fig. 4:**
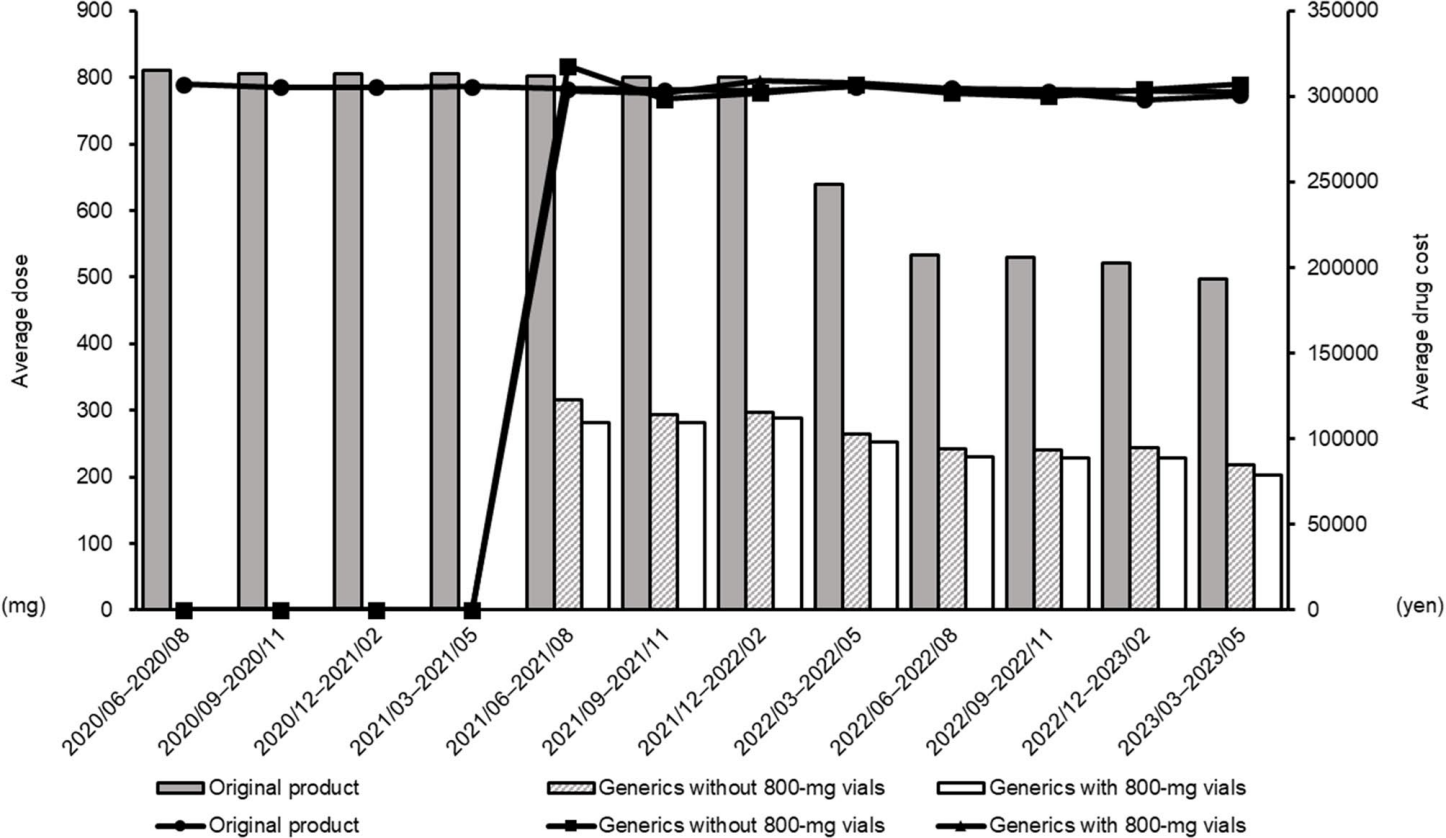
Temporal trends in average prescribed dose (potency) and average drug cost. Each data point represents an aggregate of a 3-month period (quarterly analysis). Dates on the horizontal axis are presented in the YYYY/MM (Year/Month) format

The temporal trends in mean prescribed dose (potency) and mean drug cost per prescription are shown in Fig. 4. There were no marked differences in mean prescribed dose among the three groups, with values consistently ranging between 700 and 800 mg. With respect to the mean drug cost, the cost of generics was less than 50% of that of the original product. Furthermore, from June 2022 onward, among generics, those containing 800-mg vials were approximately 4,000 to 5,000 yen less expensive per prescription compared with the generics without 800-mg vials.

The mean number of vials per prescription, calculated from the total number of vials and total number of prescriptions during the study period, is listed in Table 3. The mean number of vials was 3.4 for the original product and 3.5 for the generics without 800-mg vials, whereas it was 2.3 for the generics with 800-mg vials. Thus, the mean number of vials per prescription was approximately one fewer for generics with 800-mg vials than for the other groups.

**Table 3:** Comparison of the number of vials used by drug

	Total Number of Prescribed Vials (vials)	Total Number of Prescriptions (prescriptions)	Average Number of Vials per Prescription (vials/prescription)
Original product	191,988	56,106	3.4
Generics without 800-mg vials	8692	2454	3.5
Generic drug 1	580	167	3.5
Generic drug 2	101	36	2.8
Generic drug 4	8011	2251	3.6
Generics with 800-mg vials	70,189	30,518	2.3
Generic drug 6	12,165	4233	2.9
Generic drug 7	1	1	1.0
Generic drug 9	57,159	25,989	2.2
Generic drug 10	864	295	2.9

The results of the cost-saving simulation are as follows. Under a 100% substitution scenario within the study population from June 2022 to May 2023, switching all prescriptions from the original product to generics without 800-mg vials was estimated to reduce annual drug expenditure by 3,032,788,705 yen. In contrast, switching to generics with 800-mg vials was estimated to result in a reduction of 3,173,901,555 yen. This indicates that the use of generics with 800-mg vials may yields an additional annual saving of 141,112,850 yen compared with the use of generics without 800-mg vials.

## Discussion

In this study, we analyzed clinical data provided by multiple medical institutions to Medical Data Vision Co., Ltd. to determine the changes in prescription patterns and the effect on drug costs associated with the generic PEM in real-world clinical practice in Japan. Following the introduction of generic PEM, the proportion of generic prescriptions among PEM users showed a steady increasing trend, with particularly high prescription counts for generics that offer 800-mg vials or liquid formulations, which were not available in the original product. Moreover, the use of generics with 800-mg vials was associated with lower drug costs. As the number of vials required for preparation was smaller, the use of CSTDs could potentially be reduced based on the decrease in the number of vials per prescription. To our knowledge, this is the first study to evaluate the impact of the introduction of generic PEM on prescription volumes and drug costs across multiple products and medical institutions in routine clinical practice in Japan. Notably, by utilizing a fixed cohort of institutions that provided data continuously throughout the study period, our findings reflect shifts in prescribing behavior while minimizing potential biases that could arise from temporal changes in the number of institutions participating in the database.

During the study period, generics with 800-mg vials (by strength) and liquid formulations (by dosage form) were prescribed in greater numbers at medical institutions compared with other generics, which was accompanied by a higher number of patients and prescriptions. According to the manufacturer, among generics without 800-mg vials, “Generic drug 4” was most frequently prescribed, whereas among generics with 800-mg vials, “Generic drug 9” accounted for the highest prescription volume. These results suggest that the overall results of this study were largely affected by the prescription volumes of these products. According to the 9th National Database of Health Insurance Claims and Specific Health Checkups of Japan (NDB) Open Data released by the Ministry of Health, Labour, and Welfare (covering claims from April 2022 to March 2023), these products ranked among the top in terms of the number of prescribed vials for PEM formulations, and our study demonstrated a consistent trend [10]. The advantage of prescribing 800-mg vials is evident from the reduction of the number of vials required when a dose exceeding 700 mg is required. Moreover, the use of liquid formulations eliminates the need for reconstitution, thereby simplifying the preparation process. Previous studies have shown that the use of a generic product marketed by Nippon Kayaku Co., Ltd. (liquid formulations with 800-mg vials) reduced the drug preparation time [5]. Based on these results, prescribing generic formulations with strengths or dosage forms not available from the original manufacturer may provide operational advantages. These products may have been perceived by medical institutions as having added value, which could partly explain their higher prescription volumes. Regarding the patient characteristics by drug, for all formulations, the proportion of male patients was higher, and patients aged 60–70 years accounted for approximately 70–80% of the total. National surveys by the Ministry of Health, Labour, and Welfare indicated that the incidence of lung cancer is higher in men, with a marked increase in incidence above the age of 60 [11]. The results of our study revealed a similar trend.

When the study drugs were classified into three groups, including the original product, generics without 800-mg vials, and generics with 800-mg vials, the number of patients and prescriptions for generics showed an increasing trend over time following their market entry, with generics containing 800-mg vials showing the largest increase. Regarding the distribution of prescribed vial strengths, the proportion of 100-mg and 500-mg vials for the original product and generics without 800-mg vials were lower compared with that of the generics with 800-mg vials. In contrast, for generics with 800-mg vials, the proportion accounted for approximately 40–50% of all prescriptions by the end of the study period. This indicates that for nearly half of all prescriptions for these generics, 800-mg vials were used, which likely contributed to a reduced frequency of 100-mg and 500-mg vial prescriptions. This increase in absolute prescription counts within the fixed institutional cohort primarily reflects the transition from the original product to generic alternatives among patients receiving PEM therapy.

Regarding the average prescribed dose per case, all three groups, including the original product, generics without 800-mg vials, and generics with 800-mg vials, remained within the range of 700–800 mg, with no marked differences observed among the groups. In contrast, the average drug cost per prescription for the generics was less than 50% of that for the original product. Furthermore, generics with 800-mg vials were less expensive compared with those without 800-mg vials. As demonstrated in our simulation results, this was associated with a greater cost-reduction effect for generics with 800-mg vials than for those without. This difference was attributable to two factors: (1) during the study period, the official drug prices of generics were set at less than 50% of those of the original product, and (2) the per-milligram price of the 800-mg vial was lower compared with that of the 100-mg and 500-mg vials. Between June 2022 and May 2023, the average drug cost of the generics with 800-mg vials was approximately 4,000–6,000 yen less per prescription compared with the generics without 800-mg vials. Regarding the total annual drug expenditure, our simulation results demonstrated that switching to generics with 800-mg vials provides a greater cost-reduction effect than switching to those without. Consequently, the additional annual saving of approximately 141 million yen estimated within our study population highlights the potential economic advantage of adopting high-dose vial formulations.

In terms of the average number of vials per prescription, calculated from the total number of vials and total prescriptions during the study period, generics with 800-mg vials required approximately one fewer vial per prescription compared with the original product and generics without 800-mg vials. PEM is classified as an HD, and the use of a CSTD is recommended during preparation [3]. In Japan, when aseptic compounding is conducted using a CSTD, an additional reimbursement fee can be claimed; however, this fee is fixed regardless of the number of CSTDs used. As a result, an increased number of CSTDs results in a greater financial burden for medical institutions, and from a management perspective, minimizing CSTD use is preferable. The results of this study suggest that the use of generics with 800-mg vials reduces the number of vials required for compounding, thereby potentially contributing to a reduction in CSTD use. This, in turn, could alleviate the economic burden on medical institutions. Between June 2022 and May 2023, switching all prescriptions from the original product to generics with 800-mg vials could reduce the annual number of vial use by approximately 32,000, which suggests a potential for a proportional reduction in CSTD consumption. Previous studies have proposed cost savings by revising the available vial sizes, and most suggested the introduction of lower-dose vials based on simulations using real-world usage data [6, 7]. In contrast, the present study confirmed, using post-marketing real-world data, that depending on the pricing strategy, high-dose vials can also result in cost savings. Moreover, for drugs such as HDs in which CSTD use is recommended, the potential for reducing CSTD consumption along with drug costs was demonstrated.

This study had several limitations, which can be categorized into three groups: database-derived constraints, methodological limitations, and the generalizability of the findings. First, regarding database-derived limitations, the results were derived solely from claims data; therefore, specific clinical parameters and institutional details could not be verified. Specifically, data were unavailable for patient body weight, actual administered dose, underlying disease, and specific billing practices at each institution (e.g., whether unused drug amounts were billed when discarded or whether split dosing was implemented). Information regarding the adopted vial sizes and the timing of their adoption was also not confirmed. Second, in terms of methodological limitations, as described in the Methods section, the system specifications of the MDV analyzer restricted our analysis to mean values rather than dose distributions per prescription. Furthermore, while we analyzed temporal trends at 3-month intervals (quarterly) to capture shifts following generic entry, this aggregation might still obscure extremely short-term changes occurring immediately after the launch. Additionally, our analysis focused on the absolute number of prescriptions among PEM users to evaluate substitution dynamics and cost impacts. Although this approach directly addresses the study’s economic objectives, it does not account for changes in the overall PEM utilization rate within the entire lung cancer population. Third, regarding the generalizability of the findings, while the MDV analyzer covers approximately 32% of acute care hospitals in Japan and represents a significant study population, it does not include clinical data from all medical institutions nationwide. Thus, caution is required when generalizing these results to the entire Japanese healthcare setting. Future studies should consider these limitations.

## Conclusions

In this study, real-world prescribing patterns and drug costs associated with the introduction of generic PEM in Japan were analyzed using claims data from the MDV analyzer^®^. After the introduction of generics, their prescription share showed an increasing trend over time, with liquid formulations and 800-mg vials—both unavailable in the original product—being prescribed more frequently. The use of generics with 800-mg vials was associated with additional reductions in cost and in the number of vials required per prescription. Furthermore, because PEM is classified as an HD and the use of a CSTD is recommended during preparation, reducing the number of vials potentially offers savings in material costs in addition to drug costs. As generics are typically launched several years after the original product, they may address the challenges that were present when only the original product was available. Reports based on real-world prescriptions before and after the launch of generics remain limited; however, the present findings provide important insight for future drug development and for achieving healthcare cost reduction, including potential savings in material costs.

## Data Availability

We used non-publicly available aggregated data obtained from the ”MDV analyzer”, a large-scale data analysis web tool provided by Medical Data Vision Co., Ltd. No raw data were accessed in this study. Medical Data Vision Co., Ltd. obtains usage permissions from approximately 572 DPC hospitals across Japan and collects de-identified administrative claims data from each institution, thereby constructing a large-scale commercial database.

## Abbreviations

PEM: Pemetrexed
NSCLC: Non–small cell lung cancer
HD: Hazardous drug
CSTD: Closed-system drug-transfer device
DPC: Diagnosis procedure combination
NDB: National database of health insurance claims and specific health checkups of Japan
